# Gender Disparity and the Association Between Socioeconomic Status, Mental Health, and the Need for Long-Term Services and Support Among the Older Koreans

**DOI:** 10.3389/fpubh.2022.888011

**Published:** 2022-06-03

**Authors:** Bo Zhao, Fanlei Kong, Dong Eun Shin, Eun Woo Nam

**Affiliations:** ^1^Department of Health Administration, Graduate School, Yonsei University, Wonju, South Korea; ^2^Yonsei Global Health Center, Yonsei University, Wonju, South Korea; ^3^Centre for Health Management and Policy Research, School of Public Health, Cheeloo College of Medicine, Shandong University, Jinan, China; ^4^National Health Commission (NHC) Key Lab of Health Economics and Policy Research, Shandong University, Jinan, China

**Keywords:** socioeconomic status, mental health, need for long-term services and support, older Korean adults, gender disparities, structural equation modeling

## Abstract

**Background:**

Population aging—the inevitable increase in the percentage of older adults—is occurring all around the world as the fertility rate declines and life expectancy rises. This study examined the relationship between socioeconomic status (SES), mental health, and the need for long-term services and support (LTSS) among Korean older adults. It also aimed to provide evidence-based information for South Korea's long-term support services and programs.

**Methods:**

This study used the data on older adults aged over 60 years from the 2018 Korean Longitudinal Study of Aging (KLoSA). Ultimately, 5,527 older adults were included in the database (42.6% men, 57.4% women). To clarify the association between SES, mental health, and the need for LTSS among older Korean men and women, chi-squared test, *t*-test, and structural equation modeling (SEM) were performed.

**Results:**

The SEM analysis showed that a significant, negative association was observed between SES and the need for LTSS among these older adults—the higher SES groups would generally have less need for LTSS. Mental health had a strong, negative impact on the need for LTSS—better mental health status of the older individuals would indicate a lower need for LTSS, and the effect was stronger among older men. Meanwhile, the positive relationship between SES and mental health was verified—the higher the SES of these older adults the better their mental health.

**Conclusions:**

Gender- and social class-sensitive impacts of mental health on the need for LTSS were observed in this study. These findings could provide an evidence-based reference for interventions targeting different genders and social classes in Korea's long-term care system, such as the enhancement of social welfare and mental health status of the older adults.

## Introduction

Driven by decreasing levels of mortality and increasing life expectancy, the world continues to witness unprecedented, sustained changes in the age structure of the global population. People live longer, and the overall population has rapidly increased, with a greater proportion of older people. All regions worldwide will experience increasing elderly populations from 2020 to 2050, as the percentage of this population aged 65 years or older could grow from 9.3 to 16.0% during that time (1). South Korea officially became an aged society in 2017, with over 14% of its people aged 65 years or older (2). As of 2020, South Korea is the country with the world's lowest total fertility rate (0.84) and first reported more deaths than births (3, 4). In South Korea, the proportion of people aged 65 years or older is estimated to rise from 15.9% in 2020 and reach 40.1% by 2060 (5, 6). Thus, South Korea's aging population has become a serious concern.

Compared with other groups, older adults exhibit “frailty”—they are physically unhealthy and mentally weaker (7). As aging progresses, their decreasing cognitive ability of them will compel them to find available care services (8). The United Kingdom, Australia, Japan, and other welfare states have become aging societies and have responded by providing community-based sustainable health-promotion services; these are integrated with healthcare and welfare based on cooperation with central and local governments (9, 10). As a super-aged society, South Korea has placed the older people as a target for various supports and services in terms of economic aspects, such as long-term care insurance and the Framework Act on Social Security laws (11). Series of long-term care-related programs and facilities have been established for older adults to assist in their daily lives to improve their quality of life since 2008, and the services providers also rapidly expanded (12).

Long-term services and supports (LTSS) comprise day-to-day help needed by people with long-term conditions, disabilities, and frailty (13). This could include self-care (e.g., bathing, dressing, and toileting), help with household tasks such as meals, transportation, bills, and complex health care (medications, wound care), and other ongoing social services (14). Older people in Korea have the highest poverty and suicide rates and medical costs among the OECD countries; however, the aging response index that measures the level of preparedness for aging is the lowest (15). Because the majority (70–80%) of the long-term care services providers and institutions are private (16), issues of concern include criticized quality, insufficient mutual cooperation, and communication that prevents sustainable services from being provided (17); further, the perception of the services and supports used is significantly lower than in other welfare countries (18). In Korea, older people accounted for about 23.5% of one-person households in 2015 and the proportion of seniors living alone increased to 33.5% in 2016 (19, 20). Moreover, from 2008 to 2016, family structure and attitudes about older people's care have changed significantly. The proportion of parents who live with their children declined from 38.0 to 29.2%; surveys indicate that the increasing Korean population thought that parental support should not only be in the family and need support from the government and society (21). However, their use and eligibility for benefits face challenges and are more limited in home-based (68%) care and support (22).

Existing research supports the widely held conclusion that the need for LTSS (informal or home care) disparities could result from socioeconomic status (SES) (23, 24). SES is a significant factor that affects older adults' LTSS preferences in the care of type (25). Earlier studies have discovered that older people with higher educational attainment and household income were less functionally affected and had more predictive attitudes and planning of LTSS (26, 27). SES most commonly encompasses income and the attainment of education and occupational prestige, which is typically the social position or class of a person or group (28, 29). As educational achievement is typically determined early in life, education is considered a sign of the first choice. Income or related working occupation characteristics might be chosen widely to investigate the links between SES and health (30). In Korea, compression of morbidity and health care utilization were more common among those with a high SES (16). Furthermore, a cross-sectional study reported that women were more likely than men to plan to need specialized LTSS, such as homecare (31).

Mental health has long been a primary topic in many research fields, especially in terms of aging and healthcare support. Meanwhile, owing to the functional decline, the need for LTSS among older adults is impacted by biomedical causes and psychological disorders (32, 33). The literature has reported that elderly people who perceive high levels of isolation or loneliness show low levels of physiological functioning' repair and maintenance (34). The increased use of medical services and frequency of physician visits are influenced by depression among the elderly in primary care practices (35, 36). Additionally, older adults who had lower life quality ratings were inclined to increase the risk of complex medical needs (37). Research on Japanese and Chinese elderly (38–40) also indicated that mental health negatively affects the need for LTSS—better mental health status corresponds to a lower need for LTSS; however, the strength of such effects also varies depending on gender and age. It has been recognized that a person's mental health and the family's wellbeing should be emphasized across healthcare and LTSS systems (41).

Previous studies have also observed a relationship between mental health and socioeconomic status (SES). These studies have consistently indicated that lower SES is linked to an increased incidence of psychiatric disorders and more persistent patterns of depression (42–44). For example, adults living in the low-income group reported an increased risk of mental health problems than individuals who live in the high-income group (45, 46). Moreover, older, less-educated individuals with longer working times are most likely to experience mental disorders and suicidal ideations in Korea (47–49). A study about the Japanese aged 65 years and older mentioned working (full-time or part-time job) played a positive role in slowing the deterioration of mental health (50). Therefore, changes in SES could lead to subsequent changes in mental health status with the impact of individual and contextual determinants (51).

Understanding the connections between socioeconomic status and older adults' wellbeing is especially critical in the pledge of the United Nations' 2030 Agenda for Sustainable Development. Its Sustainable Development Goals include an emphasis on the most disadvantaged, including elderly adults, and can be accomplished in all sectors and levels of society (52). Therefore, the associations between SES, mental health, and the need for LTSS for older people have become an increasingly important policy concern, especially in countries with populations in advanced stages of aging.

Some previous studies have mostly explored the relationship between SES and the need for LTSS, mental health, and the need for LTSS, SES, and mental health. Seldom analysis included all the three variables, let alone among older Korean adults. Traditional modeling methods that merely demonstrate correlations between variables were mainly used. Structural equation modeling (SEM) is a useful analytical method for testing and estimating associations by combining statistical data with qualitative assumptions while simultaneously accounting for measurement error (53). The paths in modeling indicate the hypothesized relationships between variables that can work as both predictors and responses simultaneously and serve as mediators between explanatory and response variables (54). Hence, to fill the research gap, this study aimed to identify the relationships between SES, mental health, and the need for LTSS among older Koreans using excessive sample data by employing SEM and further explain whether a gender disparity exists in the relationship.

## Methods

### Study Design and Data

The study used cross-sectional data from the Korean Longitudinal Study of Aging (KLoSA) Wave 7 (2018) in this study. The KLoSA was launched in 2006 and includes a nationally representative sample of Koreans aged 45 years or older obtained through multistage stratified probability sampling based on geographical area (55). The KLoSA is the first nationwide study of South Korea and was founded by the Ministry of Labor, which tests depressive symptoms and is open to the public. In interdisciplinary research on the social, economic, physical, and psychological issues related to aging, the KLoSA has also been developed to gather the basic data needed to devise and implement effective social and economic policies to address the trends that emerge as the population ages. Data were collected using computer-assisted personal interviewing with a good response rate. A total of 6,940 samples were collected in Wave 7, and the final number of the people aged over 60 years old included in this study after the screening was 5,527 (2,353 men and 3,174 women).

### Measurements

#### Assessment of SES

SES is a multidimensional concept affecting health through various components including income, education, and occupation, one of which may have different impacts on the health status and healthcare usage (56, 57). This study evaluated the educational level by asking the participants the question “Which level of education did you complete?” with five options: (1) elementary school and below, (2) middle school, (3) high school, or (4) college/university or higher. Income is closely related to employment, and household income could be a good alternative to assess the older individuals' SES (30). Annual household income (unit: KRW: 10,000 won) was discretized based on quartile: (1) Q1: ≤ 1,000 (USD 8,500), (2) Q2: 1,001–2,000 (USD 17,000), (3) Q3: 2,001–3,500 (USD 29,750); (4) Q4: >3,500. According to the concept of productive aging, working at a later age is a healthy approach to maintaining one's health (50). More questions were asked to these older adults, such as “current work or not”. The answers including (1) “yes” or (2) “no” were included in this study.

#### Assessment of Mental Health

Many interdisciplinary studies have recognized and researched the concept of mental health as a primary component of wellbeing (58). This study assessed the mental health of older adults by 3 dimensions (40, 59) and asked them three questions (1) “How satisfied are you with your health status?” (subjective health); (2) “How satisfied are you with your overall quality of life?” (life satisfaction); (3) “How about your feelings and behaviors during the past week?” (past week mental health: reversal of past week depression). Responses for the first two questions range from 0 (“absolutely no”) to 100 (“absolutely certain”), and responses were classified into two categories: good (scores > 50) and bad (scores ≤ 50). The third question was measured by the Center for Epidemiologic Studies Depression Scale, a 10-item Likert scale questionnaire assessing depressive symptoms in the past week (60). Options for each item range from “most of all of the time” (score of 3) to “rarely or none of the time” (score of 0). The total scores ranged from 0 to 30. Higher scores suggest a greater severity of symptoms (past week depression). The Cronbach's alpha coefficient of the scale in this study was 0.863. The Kaiser-Meyer-Olkin measure of sampling adequacy was 0.904, with a result for Bartlett's test of sphericity of <0.001.

#### Assessment of Need for LTSS

In South Korea, the eligibility for using the long-term care related service and supports for older adults to assist in their daily lives is divided by a set of government-issued criteria and checklists, including the fundamental assessment of activities of daily life disabilities and functional limitations (61). The need for care could be reflected in the phases of the caregiving trajectory by episodic status or events such as pains and hospitalization (14). In this study, the assessment of the need for LTSS by four variables. (1) “total ADL/IADL”: Activities of daily living (ADL) and Instrumental Ability of Daily Life (IADL)were measured in this survey using 17 questions, with 3-level answers (1 = do not need help, 3 = need help in every respect). (2) Activity limitation by health (1 = not at all, 4 = very much); (3) hospitalization times in the past 2 years; (4) the number of body pains (head, shoulder, arm, fingers, etc., 13 pains in total).

### Statistical Analysis Approach

Descriptive statistics were collected to describe the participants' demographic characteristics. The gender disparity in social-demographic characteristics, SES, mental health indicators, and the need for LTSS indicators were measured using a chi-squared test. A *p*-value of <0.001 denotes statistical significance, and all the analyses were performed using the Statistical Package for Social Science (SPSS) software suite, version 24.0.

SEM was performed to investigate the relationship between SES, mental health, and the need for LTSS in older Koreans. SEM is expressed by path diagrams, which allow the researcher to diagram the hypothesized set of relationships in the model. Factors have two or more indicators called latent variables, which are represented using circles or ovals in path diagrams. The model in the SEM consists of two types of latent variables: exogenous variables (determined outside the model) and endogenous variables (determined by the model).

In the study, the exogenous variable was SES, and the endogenous variables were mental health and the need for LTSS. Lines indicate relations between variables; A line with one arrow represents a hypothesized direct relationship between two variables. The direction of the arrows connecting each exogenous variable to its' indicators (pointing to the indicators): the latent variable is measured by indicators. SES was measured by three variables: education level, household income, and current work. Mental health was measured based on respondents' health satisfaction, life satisfaction, and past week mental health. The need for LTSS was measured by four variables: total ADL/IADL, activity limitations, hospitalization times, and the number of pains.

The maximum-likelihood estimation to predict the best-fitting model was used in this study. The parameters were estimated in the model. To interpret and compare, regression coefficients between variables were focused on standardized regression coefficients shown in the diagrams. The AMOS version 21 statistical software package for Windows was used to perform the SEM, measure the model's fitness indexes, and obtain the maximum-likelihood estimates of model parameters.

## Results

### Sample Characteristics

Table 1 illustrates the basic descriptive demographic characteristics of older adults. Of the 5,527 participants involved in our analysis, 2,353 (42.6%) were men, and relatively fewer were women (3,174, or 57.4%), with an average age of 72.80 ± 8.55 years in 2018. Men and women showed different proportions of marital status. The percentage of older men who were married or living with a partner was higher than that of older women, while older women showed a higher proportion in the widowed/missing group. The older persons who lived alone or living with a spouse in one generation accounted for the majority, while there were differences in the proportions between male and female groups (16.0 vs. 31.5%; 54.4 vs. 37.1%). In addition, almost 70% of them were aware of the existence of long-term care services and supports, including the institutional/home care services, activity support services, etc. However, the proportion of those using in 2018 shared only 2.5% among these older Koreans.

**Table 1:** Participants' descriptive characteristics.

**Variables**	**Men**	**Women**	**Total**
	***n* (%)/mean ±S.D**.	***n* (%)/mean ±S.D**.	***n* (%)/mean ±S.D**.
Participants	2,353 (42.6%)	3,174 (57.4%)	5,527 (100%)
Age	72.17 ± 8.26	73.28 ± 8.73	72.80 ± 8.55
Marital status			
Married/living with a partner	2,124 (90.3%)	1,848 (62.0%)	3,972 (71.9%)
Separated/divorced	60 (2.5%)	74 (2.3%)	134 (2.4%)
Widowed/missing (dispersed family)	153 (6.5%)	1,240 (39.1%)	1,393 (25.2%)
Never married	16 (0.7%)	12 (0.4%)	28 (0.5%)
Households composition			
Single household	377 (16.0%)	1,001 (31.5%)	1,378 (24.9%)
Couple household/one generation	1,279 (54.4%)	1,177 (37.1%)	2,456 (44.4%)
Two generation	586 (24.9%)	712 (22.4%)	1,298 (23.5%)
Three generation or more/others	111 (4.7%)	284 (9.0%)	395 (7.2%)
Awareness of supports and services			
Yes	1,618 (68.8%)	2,157 (68.0%)	3,775 (68.3%)
No	735 (31.2%)	1,017 (32.0%)	1,752 (31.7%)
Current using supports and services			
Yes	45 (1.9%)	93 (2.9%)	138 (2.5%)
No	2,308 (98.1%)	3,081 (97.1%)	5,389 (97.5%)

Table 2 displays information regarding the descriptive characteristics for the selected variables by gender. Regarding age, older men and women participants shared similar proportions in the three groups, with the biggest number in the range 60–69 years old. In terms of education, more older males had high school education while more females achieved elementary school education. Regarding annual household income, the older men were distributed more in high-level income, and the older women were more opposite in the low-level income. Around 70% of them did not work currently, and that was more in the women group. Additionally, significant differences in age, education, annual household income, and current work were also reported between the older men and women group.

**Table 2:** Descriptive SES, mental health, and need for LTSS characteristics of participants by gender.

**Variables**	**Gender**						**χ^2^-Test (*p*)**
	**Men (*****n*** **=** **2,353)**		**Women (*****n*** **=** **3,174)**		**Total (*****n*** **=** **5,527)**		
	***n***	**%**	***n***	**%**	***n***	**%**	
Age							
60–69	993	42.2%	1,221	38.5%	2,214	40.1%	14.27 (0.001)
70–79	857	36.4%	1,145	36.1%	2,002	36.2%	
≥80	503	21.4%	808	25.5%	1,311	23.7%	
Education level							
Elementary school and below	668	28.4%	1,928	60.7%	2,596	47.0%	711.804 (<0.001)
Middle school	441	18.7%	532	16.8%	973	17.6%	
High school	851	36.2%	601	18.9%	1,452	26.3%	
College/University or higher	393	16.7%	113	3.6%	506	9.2%	
Annual household income (per 10,000 Korean won)							
Q1	537	22.8%	1,106	34.8%	1,643	29.7%	118.814 (<0.001)
Q2	545	23.2%	754	23.8%	1,299	23.5%	
Q3	561	23.8%	647	20.4%	1,208	21.9%	
Q4	710	30.2%	667	21.0%	1,377	24.9%	
Current work							
No	1,364	58.0%	2,525	79.6%	3,889	70.4%	301.879 (<0.001)
Yes	989	42.0%	649	20.4%	1,638	29.6%	
Subjective health							
Bad	900	38.2%	1,556	49.0%	2,456	44.4%	63.531 (<0.001)
Good	1,453	61.8%	1,618	51.0%	2,178	55.6%	
Life satisfaction							
Dissatisfied	655	27.8%	1,129	35.6%	1,784	32.3%	36.970 (<0.001)
Satisfied	1,698	72.2%	2,045	64.4%	3,743	67.7%	
Past week depression							
Mean ± S.D.^a^	6.24 ± 5.433		7.13 ± 5.633		6.75 ± 6.00		−5.938 (<0.001)
Activity limitation							
Not at all	327	13.9%	305	9.6%	632	11.4%	43.876 (<0.001)
Not much	1,145	48.7%	1,449	45.7%	2,594	46.9%	
Some degree	686	29.2%	1,066	33.6%	1,752	31.7%	
Very much	195	8.3%	354	11.2%	549	9.9%	
Hospitalization times							
0	2,129	90.5%	2,785	87.7%	4,914	88.9%	13.429 (0.004)
1	168	7.1%	304	9.6%	472	8.5%	
2	36	1.5%	65	2.0%	101	1.8%	
≥3	20	0.8%	20	0.6%	40	0.7%	
Number of pains							
0	1,011	43.0%	666	21.0%	1,677	30.3%	388.824 (<0.001)
1	508	21.6%	645	20.3%	1,153	20.9%	
2	449	19.1%	805	25.4%	1,254	22.7%	
≥3	385	16.4%	1,058	33.3%	1,443	26.1%	
Total ADL/IADL							
17	2,002	85.1%	2,735	86.2%	4,737	85.7%	1.301 (0.254)
≥18	351	14.9%	439	13.8%	790	14.3%	

*^a^t-test*.

Most of the older people rated their health status as “good” and were satisfied with their quality of life. There were significant differences in subjective health and life satisfaction between older men and women. Additionally, the mental health scores of the two groups showed different levels, with higher scores in women (men: 6.24 ± 5.433, women: 7.13 ± 5.633). Concerning the activity limitation, not much limitation was reported in older men and women, and there was a high proportion of older women who reported some degree of activity limitation. Regarding hospitalization time, most of them had not used inpatient services during the past 2 years. Concerning the number of body pains, the men group showed a higher proportion of those with no pain, whereas the women group showed a higher proportion of those with multiple pains. However, there was no significant difference between men and women in the ADL/IADL scores.

### Structural Model

#### Measurement Invariance Across Gender

Table 3 illustrates the results of the selected fitness indicators regarding the measurement invariance across gender and the fitness indexes in the seven different models. The fitness indexes between the male and female elderly were first compared to demonstrate whether the gender variable is suitable for this group comparison.

**Table 3:** Model invariance test using multiple-group analysis.

**Model**	**χ^2^**	**df**	**χ^2^/df**	**GFI**	**AGFI**	**CFI**	**RMSEA**	**ΔCFI**	**ΔRMSEA**
M_1_	592.516***	64	9.258	0.979	0.964	0.933	0.039	-	-
M_2_	592.516***	64	9.258	0.979	0.964	0.933	0.039	0	0
M_3_	592.516***	64	9.258	0.979	0.964	0.933	0.039	0	0
M_4_	627.658***	71	8.840	0.978	0.965	0.929	0.038	0.004	0.001
M_5_	662.528***	74	8.953	0.977	0.965	0.925	0.038	0.004	-
M_6_	680.044***	75	9.067	0.976	0.965	0.923	0.038	0.002	-
M_7_	672.459***	77	8.977	0.976	0.959	0.922	0.038	0.001	-

*M_1_, elderly men; M_2_, elderly women; M_3_, unconstrained; M_4_, measurement weights; M_5_, structural weights; M_6_, structural covariances; M_7_, structural residuals*.

***p <0.001.

*df, degrees of freedom; AGFI, adjusted goodness of fit index; RMSEA, root-mean-square error of approximation*.

This study introduced the goodness of fit index (GFI), adjusted goodness of fit index (AGFI), and root mean square error of approximation (RMSEA) as the model fitness indexes. When the size of the samples used is sufficiently large, as in this study, a non-significant chi-squared value is rare (62, 63), which demonstrates this test's usefulness given the sample-size dependency (64). Table 4 reveals that the fitness indices of older Korean men and women were the same (or GFI = 0.979, AGFI = 0.964, CFI = 0.933, RMSA = 0.039 in both M_1_ and M_2_). The GFI, AGFI, and CFI values surpassed 0.90, and the RMSA values were <0.05, indicating that the measurement invariances between the two groups could be further explored for the other models.

**Table 4:** Standardized effects between SES, mental health, and need for LTSS by gender.

**Items**	**Direct**		**Indirect**		**Total**	
	**Men**	**Women**	**Men**	**Women**	**Men**	**Women**
SES → Need for LTSS	−0.334***	−0.347***	−0.245***	−0.255***	−0.579***	−0.601***
Mental health → Need for LTSS	−0.479***	−0.433***	-	-	−0.479***	−0.433***
SES → Mental health	−0.512***	0.588***	-	-	0.512***	0.588***

*SES, socioeconomic status; need for LTSS, need for long-term services and support*.

***p < 0.001.

We used the ΔCFI and ΔRMSEA between M_3_ (the unconstrained model), M_4_ (measurement weights model), M_5_ (structural weights model), M_6_ (structural covariance), and M_7_ (structural residuals) to evaluate the measurement invariance in this study. M_3_ does not restrict the model coefficient, while M_4_ implies that the indicator loadings for the corresponding construct are equal for each group. M_5_ constrains both the indicator loadings for the corresponding construct and structural coefficients across the groups. Conversely, the indicator loadings for the corresponding construct and the structural coefficients of each group were assumed to be equal in the M_6_. Moreover, M_7_ supposes that the indicator loadings, structural coefficients, endogenous variables' covariance, and variance of the exogenous variable across the groups are equal.

The ΔCFI between M_4_ and M_3_, M_5_ and M_4_, M_6_ and M_5_, and M_7_ and M_6_, are 0.004, 0.001, 0.002, and 0.001, respectively; ΔRMSEA is 0.001 between M4 and M3, and 0 betweenM_5_ and M_4_, M_6_ and M_5_, and M_7_ and M_6_. Previous literature states that a change of <0.010 in the CFI (ΔCFI) signifies that a measurement invariance has formed across groups (65). Regarding the ΔRMSEA, changes of <0.015 in the RMSEA (ΔRMSEA) are accepted when establishing the measurement invariance if the sample size is more than 300 (66). As all ΔCFI values in this study were <0.010 and all ΔRMSEA values were <0.015, the measurement invariance was established between the M_1_, M_2_, M_3_, M_4_, M_5_, M_6_, and M_7_ models across both the older men and women groups in this study.

#### Model Fitness Indices

**Figure 1:**
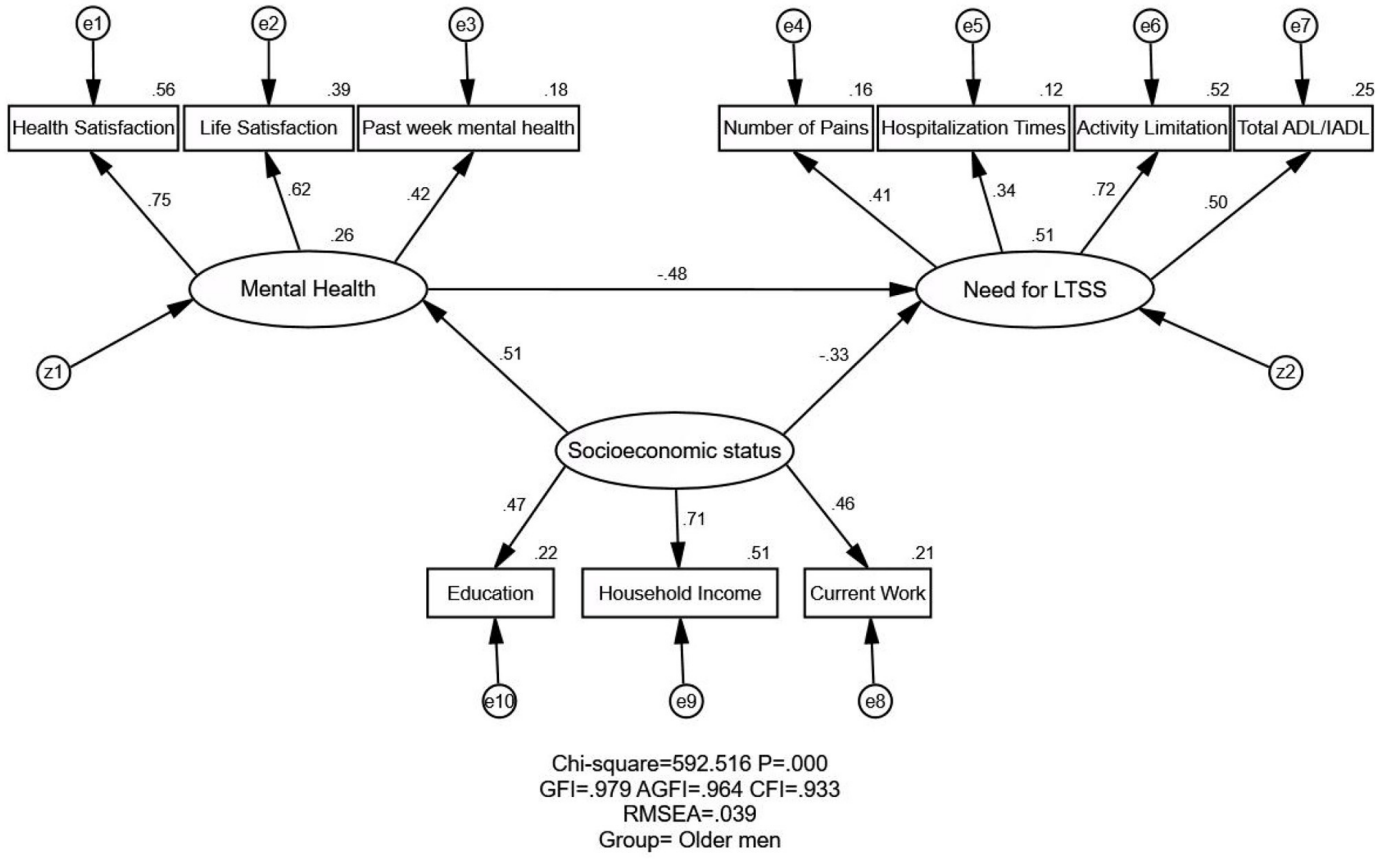
Structural equation modeling analysis of the association between SES, mental health, and the need for LTSS among South Korea's older men (*n* = 2,353). The data were employed to analyze the association between SES, mental health, and the need for LTSS. The arrows indicate the associations and directions between variables. All parameter estimates were statistically significant (*p* < 0.001). χ^2^, chi-square value; GFI, goodness of fit index; AGFI, adjusted goodness of fit index; RMSEA, root-mean-square error of approximation; SES, socioeconomic status; Need for LTSS, need for long-term services and support; CMIN, the chi-square value as noted in AMOS.

**Figure 2:**
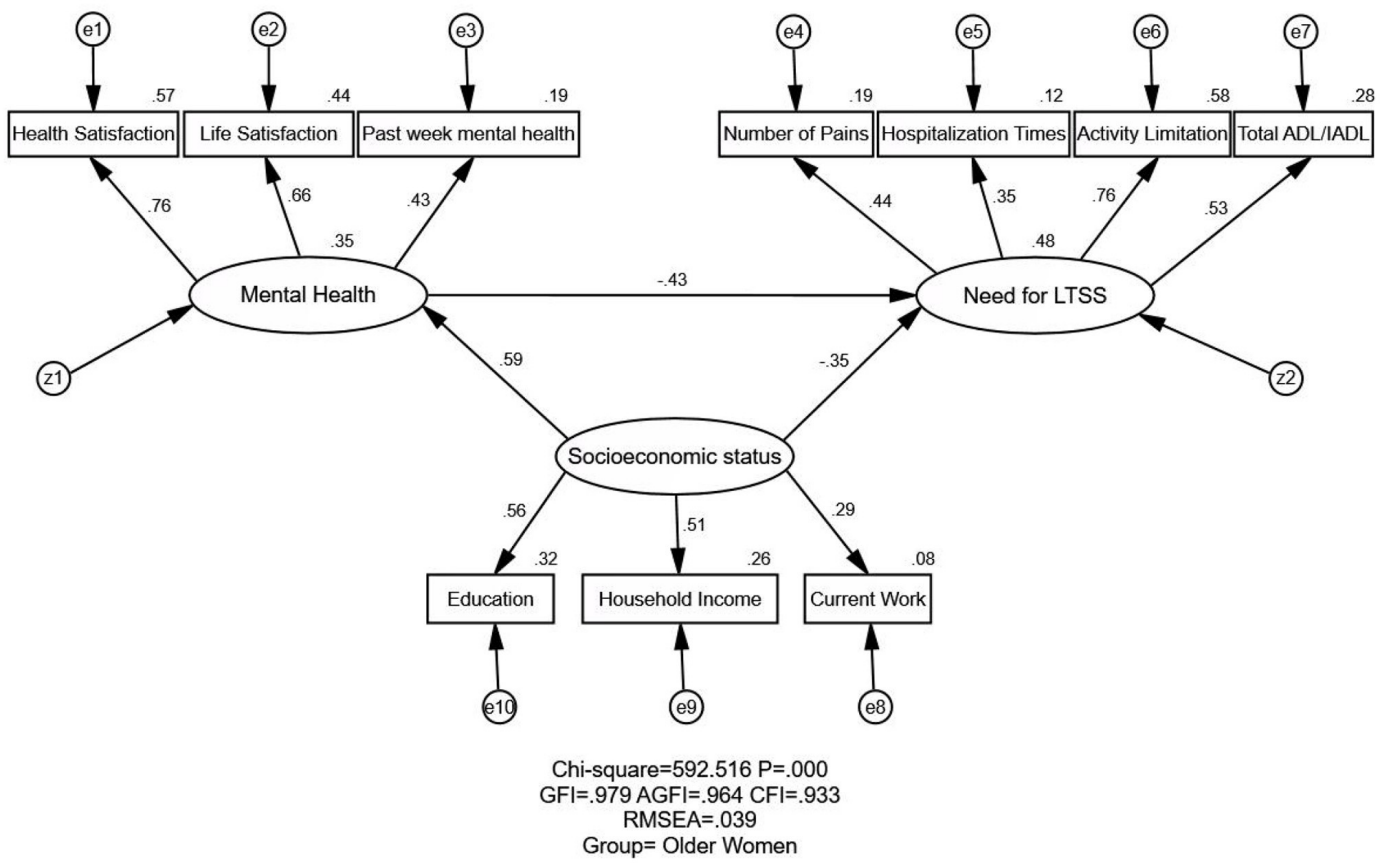
Structural equation modeling analysis of the association between SES, mental health, and the need for LTSS among South Korea's older women (*n* = 3,174). The data were employed to analyze the association between SES, mental health, and the need for LTSS. The arrows indicate the associations and directions between variables. All parameter estimates were statistically significant (*p* < 0.001). χ^2^, chi-square value; GFI, goodness of fit index; AGFI, adjusted goodness of fit index; RMSEA, root-mean-square error of approximation; SES, socioeconomic status; Need for LTSS, need for long-term services and support; CMIN, the chi-square value as noted in AMOS.

Figures 1, 2 illustrate the proposed (unconstrained) model for the older men and women groups, respectively. The estimates of model fitness were the same for the two groups: GFI = 0.979 > 0.90, AGFI = 0.964 > 0.90, CFI = 0.933 > 0.90, and RMSEA = 0.039 < 0.05. Therefore, the proposed model fits the empirical data well for both groups. However, the CMIN value (the chi-squared value in AMOS) was significant (*p* < 0.001; Table 3). As this value is sensitive to sample size, it was not used to appraise the fitness index in our study, as in prior research.

### Relationship Between SES, Mental Health, and Need for LTSS Using SEM

Table 4 and Figures 1, 2 illustrate the association and standardized effects between SES, mental health, and the need for LTSS. As mentioned in the Methods, the standardized effect is the standardized estimate path coefficients in equivalent units (range: 0–1). The total effects in the path mode can be divided into two parts: direct and indirect. The direct effect is the direct empirical association of an independent variable on a dependent variable, while the indirect effect is the indirect empirical association of an independent variable on a dependent variable through other mediators. As shown in Figure 1 or Figure 2 of the manuscript, SES → need for LTSS shows the direct effect from SES to the need for LTSS. While SES → Mental health → need for LTSS illustrates the indirect effect of SES on the need for LTSS through Mental health.

#### Relationship Between SES and Need for LTSS

The relationship between SES and the need for LTSS has been reported as negative, direct, and indirect (through mental health). The negative and direct effects of SES on need for LTSS were observed in both older men (standardized direct effects = −0.334) and women (standardized direct effects = −0.347). Meanwhile, SES negatively and indirectly affected the need for LTSS through mental health (standardized indirect effects = −0.245 for older men and −0.255 for older women). Ultimately, SES exerted a stronger negative effect on the need for LTSS for older women (standardized total effects = −0.601 for men and −0.579 for women), indicating that older women with higher SES generally have less need for LTSS.

#### Relationship Between Mental Health and Need for LTSS

A direct negative relationship was observed between mental health and the need for LTSS among the two groups. The better the mental health, the less the need for LTSS, indicating that improving mental health among the elderly would reduce the need for LTSS. Additionally, the relationship between mental health and the need for LTSS was higher for older men (standardized direct/total effect = −0.479) than older women (standardized direct/total effect = −0.433).

#### Relationship Between SES and Mental Health

The SES's direct and positive effects on mental health were shown among the men and women groups. Therefore, the higher the level of SES among these older adults, the fewer mental disorders, and the better their mental health. Each group exhibited statistical significance, and the coefficient was stronger in older women (standardized direct/total effect = 0.588 for older women and 0.512 for older men).

## Discussions

### Summary of the Findings

#### SES and Need for LTSS

This research has noted the negative effect of SES on the need for LTSS in both Korean older men and women. Similar findings have been observed in elderly populations in Canada (67), Vietnam (68), Japan (38), and China (40). These studies have identified that socioeconomic factors, such as education, household income, and work status, are related to the need for LTSS. This finding may explain why earlier research has demonstrated that people who live in the most disadvantaged regions report higher levels of unsatisfactory long-term care needs (69). Notably, although the association between SES and the need for LTSS was found among both men and women, SES exerted a slightly stronger effect on the need for LTSS for older women than for men. This result was in line with a previous study on Chinese older adults, which stated that socioeconomic factors were stronger among women for the use of LTSS (40). This may be due to the women's older average age and a greater proportion of living alone, resulting in a higher level of dependence on socioeconomic and care support (70). Additionally, as for the SES indicators in the model, education, income, and work showed different effects in the men and women groups. In the 1960s and 1980s, women were gradually integrated into the labor and educational system (71). Thus, household income and work had stronger effects in the men group, while the effect of education in the women group was stronger. It may be inferred that the Korean women who need care could receive more help from their spouses and children (72).

#### Mental Health and Need for LTSS

The results of this study demonstrated the negative relationship between mental health status and the demand for LTSS in gender groups. This parallels the results of previous research conducted on senior citizens in Japan and China (38–40). The older men got lower scores on the depression scale and had a better level of mental health (men: 6.24 ± 5.433; women: 7.13 ± 5.633). However, the men's dysfunctional coping strategies for mental distress, such as overusing alcohol and reducing social connectedness, expose them to various undesirable physical or mental outcomes that rely more on help from their spouse or family and get more effect from mental health fluctuations on the need for LTSS (73). From the standpoint of healthcare professionals, older persons with mental disorders could suffer exhaustion and distancing; simultaneously, there is the inference of a possibility of blaming even abandonment (74). According to the previous study (39), older individuals in Japan who were more socially isolated did not prefer getting care services at their own or relatives' homes even when they required care. Therefore, recognizing the role of such emotional and mental health problems such as depression—in the case of older people—in reducing people's ability and motivation to manage their health is critical to avoiding unmet LTSS (75, 76).

#### SES and Mental Health

The strong positive association between SES and mental health is compatible with previous research: (1) the population with lower SES appears to have more mental health issues (44), and (2) those with higher SES states are typically better off (77, 78). The positive relationship between SES and mental health observed in this study differed by gender, which was stronger in older women. Previous studies have reported the differential effects of sub-indicators on mental health. Income and work status could lead to stronger relationships with SES in older men than women, while education is more strongly related to SES in women than in men (79). Women who own less SES would matter less, that it would be more dependent on their spouses' SES, and older women's social status might be more reliably measured by their educational history (80, 81). Additionally, when facing dynamic economic status changes, older women were reported more likely to have a continuously high level of depression than older men (82). This could support the hypothesis that SES indicators are differently associated with mental health in men and women.

#### SES, Mental Health, and Need for LTSS

This study explored the relationship between SES, mental health, and the need for LTSS using SEM among older Korean adults. A significant negative association was observed between SES and the need for LTSS among older adults. Mental health had strong negative impacts on the need for LTSS in both gender groups, with a higher level in older males. Meanwhile, SES was also significantly and positively associated with mental health for both genders, and this was a little stronger among older females.

SES inequality could contribute to different levels of mortality, behavioral risk factors, and psychological stress (83, 84). In examining previous studies (38, 40), the current findings can be well explained as follows. South Korea's older adults from different SES levels live in different communities with different environments—the disparity in SES could imply a different level of access to health services. These environmental community factors also lead to different mental outcomes among older persons; when their mental health deteriorates, the need for LTSS progressively appears and rises. Therefore, there were different effects of SES indicators on mental health and the need for LTSS.

Numerous studies have already examined the relationship between SES and health to reveal significant associations consistently in this relationship. Typically, this has suggested that SES was associated with health outcomes in different races and the treatment choice in healthcare (85, 86), Although gender-based discrimination in employment and education has somewhat changed throughout South Korea's economic development process (87), women in South Korea still have a lower SES than men and a higher prevalence of depression and chronic diseases even today (88), Moreover, in the traditional family model, men are expected to be primarily responsible for economically supporting the household, while women are expected to leave the labor force and care for children at home as families form and grow. Notably, this seems to have more profoundly influenced Korean family characteristics with fewer satisfactory marriages and less egalitarian divisions of housework between spouses (89), Therefore, older men may experience high levels of life dependence on their spouse with more negative mental responses over a long duration (90). Conversely, older women seem to have a higher level of need for LTSS related to chronic diseases and functional limitations.

### Limitations and Future Research

This study had several limitations. First, only one-wave data from the KLoSA survey were used in this study although it was a relatively large sample size, which could limit the generalizability of the results. The cross-sectional nature of data restricted the examination of cause-effect relationships among these factors. So, it would be planning to use the multiple waves data to explore the changes in the need for LTSS associated with SES and mental health.

Second, limited indicators for each latent variable (such as SES, the need for LTSS) are available in the SEM hypothesis model because of the questionnaire's simplified content design, and its consequent responses. Since only the gender difference of the hypothesized model was explored in this study, more research focusing on the difference in employment status, rural and urban, etc. are worthy of conducted in the future. The health of status of people at different SES levels may be impacted by multiple determinants, such as the existed physical limitation level, actual support, subjective/official assessment of the need for LTSS, or the reverse causality relationship. Meanwhile, the need for LTSS affected by age and the differences in marital status is also important for further subanalyses. Thus, further studies should consider more comprehensively the relationship between SES and health status.

Third, this study's indicators of mental health include “subjective health,” “life satisfaction,” and “prior week's depression”. These questions primarily focused on cognition and finished with self-judgment, ignoring the effects of other stressors. Subjective health may be misunderstood as the physical health-related questions by older adults. Additionally, women tend to be less optimistic about their ADL functional limitations. Therefore, a future study is planned to clarify the association between SES, mental health, and the need for LTSS using more SEM indicators.

## Conclusions and Implications

Despite some limitations, gender- and class-sensitive impacts of mental health on the need for long-term care services and support were emphasized in this study. These findings may provide an evidence-based reference for interventions targeting different genders in Korea's long-term care system.

The critical impact of SES on mental health and the need for LTSS suggests that social and political measures to improve the SES of older adults are needed, particularly in the welfare areas of the labor market and social security. Public policy that promotes early intervention and prevention for vulnerable older adults and their families should also be encouraged, as this may advance their SES and minimize SES inequality in a more effective way.

It is essential to accommodate the diversity of older people and enhance care options with personalized care arrangements. In South Korea, policy and clinical decision-making could aim to augment special care according to the severity of the disease, chronic pain, or comorbidities' effects on the health and functional status of older female patients (91). Additionally, older Korean men who have negative coping strategies for mental distress and highly depend on spouses in daily life (69) may require considerably more access to life support and mental health response. Therefore, considering the target population's characteristics and setting the priority on universal LTSS accessibility should be more focused in the future (16).

## Data Availability Statement

The data for this study were taken from the Korean Longitudinal Study of Aging (https://survey.keis.or.kr/eng/klosa/klosa01.jsp) and analyzed here. Data are available in the public domain.

## Ethics Statement

This was a secondary analysis of the KLoSA study which involved human participants. Participants provided their written informed consent to participate in this study. Participants approved by the KLoSA data were anonymized. This study has been approved by the Yonsei University Institutional Review Committee (Task No. 1041849-202104-SB-068-01).

## Author Contributions

BZ: conceptualization, methodology, formal analysis, writing—original draft, review, and editing. FK and EN: conceptualization, methodology, review, and editing. DS: review and editing. All authors contributed to the article and approved the submitted version.

## Funding

This study was supported and funded by the National Natural Science Foundation of China (grant number: 71804094) and China Postdoctoral Science Foundation (grant number: 2016M592161).

## Conflict of Interest

The authors declare that the research was conducted in the absence of any commercial or financial relationships that could be construed as a potential conflict of interest.

## Publisher's Note

All claims expressed in this article are solely those of the authors and do not necessarily represent those of their affiliated organizations, or those of the publisher, the editors and the reviewers. Any product that may be evaluated in this article, or claim that may be made by its manufacturer, is not guaranteed or endorsed by the publisher.
